# The effect of time since stroke, gender, age, and lesion size on thalamus volume in chronic stroke: a pilot study

**DOI:** 10.1038/s41598-020-76382-x

**Published:** 2020-11-24

**Authors:** Lisa C. Krishnamurthy, Gabriell N. Champion, Keith M. McGregor, Venkatagiri Krishnamurthy, Aaminah Turabi, Simone R. Roberts, Joe R. Nocera, Michael R. Borich, Amy D. Rodriguez, Samir R. Belagaje, Rachael M. Harrington, Michelle L. Harris-Love, Stacy M. Harnish, Jonathan H. Drucker, Michelle Benjamin, M. Lawson Meadows, Lauren Seeds, Zvinka Z. Zlatar, Atchar Sudhyadhom, Andrew J. Butler, Amanda Garcia, Carolynn Patten, Jonathan Trinastic, Steven A. Kautz, Chris Gregory, Bruce A. Crosson

**Affiliations:** 1 Center for Visual and Neurocognitive Rehabilitation, Atlanta VA Health Care System, 1670 Clairmont Rd, Decatur, GA 30033 USA; 2 Department of Physics and Astronomy, Georgia State University, Atlanta, GA USA; 3 Center for Advanced Brain Imaging, Georgia State University and Georgia Institute of Technology, Atlanta, GA USA; 4 Department of Psychology, Georgia State University, Atlanta, GA USA; 5 Department of Neurology, Emory University, Atlanta, GA USA; 6 Department of Rehabilitation Medicine, Emory University, Atlanta, GA USA; 7 Center for Brain Plasticity and Recovery, Georgetown University, Washington, DC USA; 8 Department of Speech and Hearing Science, Ohio State University, Columbus, OH USA; 9 Department of Physical Therapy, Brooks Rehabilitation Center, Jacksonville, FL USA; 10 Department of Psychiatry, University of California San Diego, La Jolla, CA USA; 11 Brigham and Women’s Hospital, Dana Farber Cancer Institute, Harvard Medical School, Boston, MA USA; 12 School of Health Professions, University of Alabama Birmingham, Birmingham, AL USA; 13 Clinical and Health Psychology, University of Florida, Gainesville, FL USA; 14 Department of Physical Medicine and Rehabilitation, University of California Davis, Sacramento, CA USA; 15 Data Science, Duke Energy, Charlotte, NC USA; 16 Ralph H. Johnson VA Medical Center, Charleston, SC USA; 17 Department of Health Sciences and Research, Medical University of South Carolina, Charleston, SC USA

**Keywords:** Stroke, Neurology

## Abstract

Recent stroke studies have shown that the ipsi-lesional thalamus longitudinally and significantly decreases after stroke in the acute and subacute stages. However, additional considerations in the chronic stages of stroke require exploration including time since stroke, gender, intracortical volume, aging, and lesion volume to better characterize thalamic differences after cortical infarct. This cross-sectional retrospective study quantified the ipsilesional and contralesional thalamus volume from 69 chronic stroke subjects’ anatomical MRI data (age 35–92) and related the thalamus volume to time since stroke, gender, intracortical volume, age, and lesion volume. The ipsi-lesional thalamus volume was significantly smaller than the contra-lesional thalamus volume (t(68) = 13.89, p < 0.0001). In the ipsilesional thalamus, significant effect for intracortical volume (t(68) = 2.76, p = 0.008), age (t(68) = 2.47, p = 0.02), lesion volume (t(68) = − 3.54, p = 0.0008), and age*time since stroke (t(68) = 2.46, p = 0.02) were identified. In the contralesional thalamus, significant effect for intracortical volume (t(68) = 3.2, p = 0.002) and age (t = − 3.17, p = 0.002) were identified. Clinical factors age and intracortical volume influence both ipsi- and contralesional thalamus volume and lesion volume influences the ipsilesional thalamus. Due to the cross-sectional nature of this study, additional research is warranted to understand differences in the neural circuitry and subsequent influence on volumetrics after stroke.

## Introduction

The thalamus acts not only as a relay from the periphery to the cortex, but also as a higher order relay from one cortical area to another^1^. In the context of these higher order relays, the role of the thalamus has been implicated as a platform to stabilize signals in various cortical processing streams^2,3^ and is particularly vulnerable to diaschisis after stroke, the effects of which remain poorly understood. Recent stroke studies have shown that the ipsi-lesional thalamus longitudinally and significantly decreases after stroke in the acute and subacute stages, but the contra-lesional thalamus remains relatively unchanged^4,5^. However, additional considerations require exploration including time since stroke in the chronic stages, gender, intracortical volume, aging, and lesion volume to better characterize thalamic differences after cortical infarct.

In the context of additive factors that may influence thalamus volume, it is well accepted that gender and intracortical volume influences the quantification of brain volumetrics^6^, including thalamus volume and lesion volume. Further, neuroimaging studies of the human brain have shown that thalamic volume reduces with age and is associated with aging-related cognitive and motor differences^7,8^. These studies found a direct relationship between decrease in thalamic volume and decreased density of thalamo-cortical projections, providing evidence that aging-related cortical atrophy could explain differences in thalamic volume. Thus, if gender and intracortical volume differences and aging-related atrophy have effects on the thalamus volume in healthy cohorts, then it is important to understand how stroke’s effect on thalamus volume relates to time since stroke, gender, intracortical volume, age, and lesion size to accurately design rehabilitation treatments based on these clinical factors. In this pilot study we investigate these effects on both ipsilesional and contralesional thalamus volume. The hypotheses for this cross-sectional cohort are: (1) that we will identify a negative relationship between total thalamus volume and time since stroke, (2) that gender effects on thalamus volume will be evident in both contra- and ipsilesional thalamus, (3) that age has a negative relationship with thalamus volume, and (4) that lesion volume will have a negative relationship with ipsi-lesional thalamus volume but no relationship with contra-lesional thalamus volume.

## Methods

### Subjects

This retrospective cross-sectional study combined deidentified T1-weighted (T1w) anatomical imaging data from three sites in accordance with relevant guidelines and regulations approved by the joint Atlanta VA/Emory University institutional review board, University of Florida institutional review board, or Georgetown University institutional review board. All participants provided informed consented to participate in a magnetic resonance imaging (MRI) study that incorporated a T1w anatomical image. A total of 69 chronic stroke participants (5–207 months post-stroke, 28 Female, 41 Male, age = 35–92, and lesion volume = 0.25–172 cc) were identified to have usable T1w images, relevant demographic information, and intact thalami in both hemispheres. Either the left or right hemisphere was lesioned in each participant, but not both.

### MRI processing

**Figure 1:**
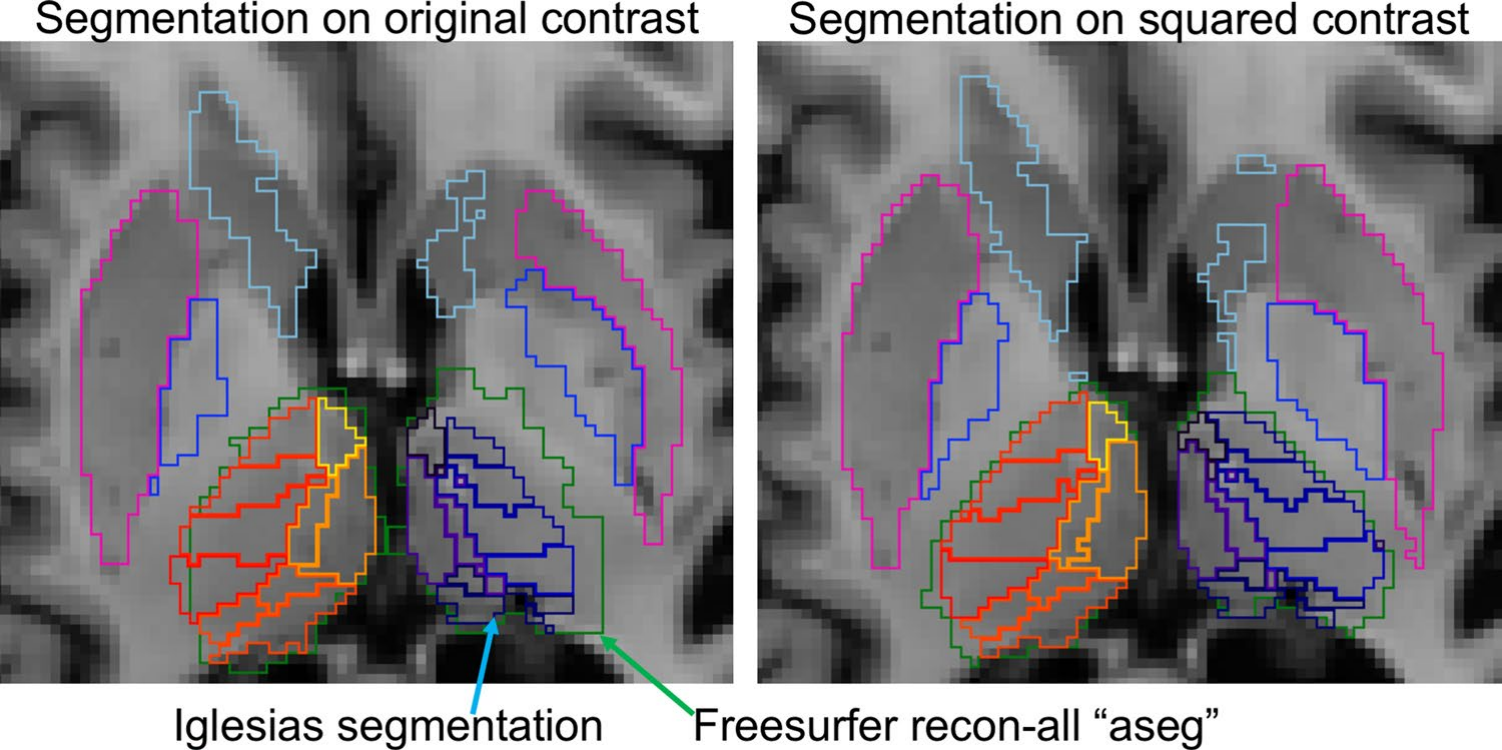
Representative freesurfer recon-all segmentation and Iglesias segmentation on original T1w contrast and squared T1w contrast.

T1w images were inspected for motion artifacts, denoised^9,10^, bias field corrected^11^, and then squared to increase the signal contrast between grey and white matter, thereby improving segmentation results of the subcortical regions. The squared T1w image was processed through Freesurfer’s cross-sectional ‘*recon-all*’ pipeline^12^ followed by the Iglesias thalamic nuclei segmentation^13^. In effect, the thalamus segmentation was optimized with a two-pronged approach: (1) by squaring the T1w signal to improve signal contrast and (2) by applying the Iglesias segmentation algorithm which uses Bayesian inference initialized by the location of the neighboring subcortical brain structures (Fig. 1).

Automatic stroke lesion segmentations were generated using LINDA^14^ on the non-squared T1w images, followed by manual touch-up with itksnap^15^ to exclude healthy areas falsely identified as lesion and to include damaged regions falsely identified as non-lesioned. For each individual subject, the thalamus volume and lesion volume were extracted for further statistical tests. The thalamus is designated as either ipsi-lesional (same hemisphere as the lesion) or contra-lesional (opposite side to the lesion) to allow combination between left and right hemisphere stroke participants. A paired student’s t-test is used to determine if contra- and ipsilesional thalamus volume are significantly different.

### Model fit of time since stroke, gender, intracortical volume, age, and lesion volume

We completed an ANOVA in JMP Pro15 to test if (time since stroke), (gender), (intracortical volume), (age), (lesion volume), and cross terms (time since stroke*age), (time since stroke*lesion volume), (intracortical volume*lesion volume), (gender*intracortical volume), and (age*lesion volume) explain ipsilesional and contralesional thalamus volume. The results of the model are reported with F-statistic and subsequent t-tests are performed to determine which terms had significant effect.

## Results

**Figure 2:**
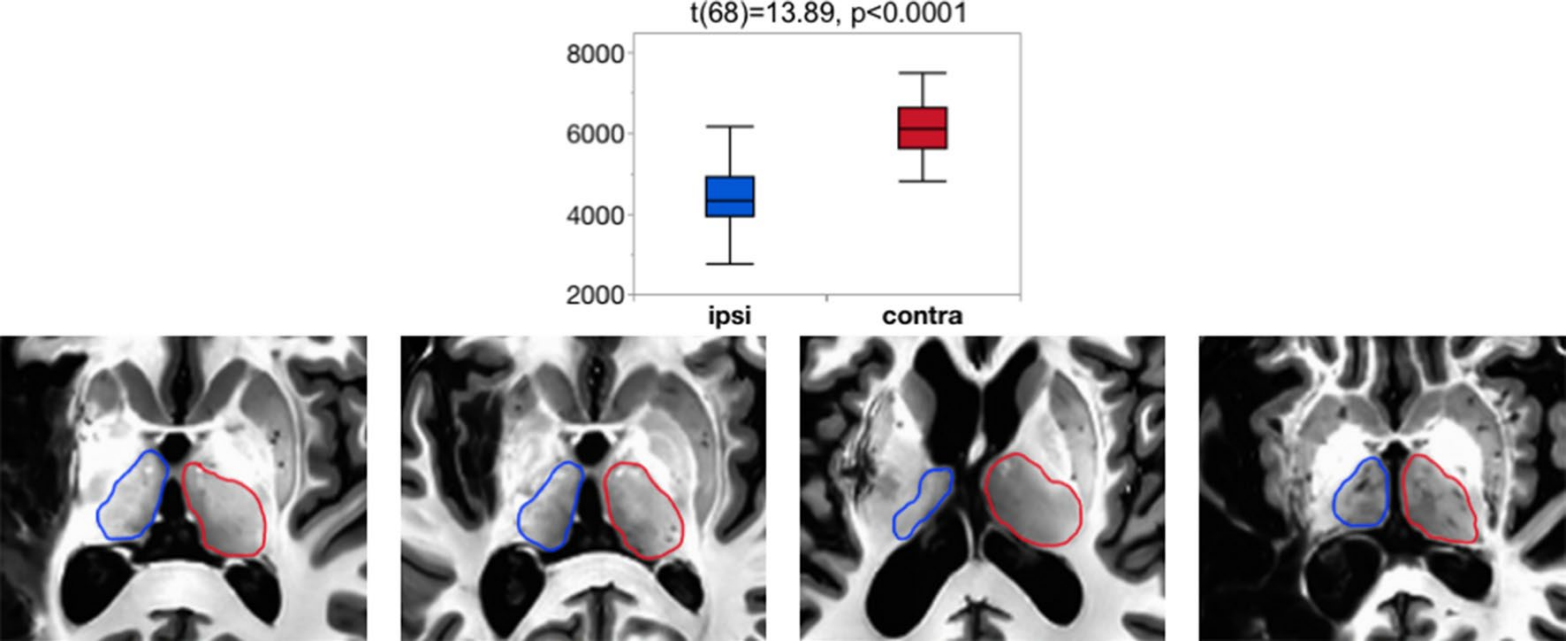
Ipsilesional thalamus volume is significantly smaller than contralesional thalamus volume. This effect can be identified visually from the T1w images. Note: blue = ipsilesional thalamus, red = contralesional thalamus.

The ipsi-lesional thalamus volume was confirmed to be smaller than the contra-lesional thalamus volume (t(68) = 13.89, p < 0.0001) using a two-tailed paired Student’s t-test in JMP Pro15. These effects can be identified in the T1w images with visual inspection, especially in the participants with larger lesions (Fig. 2).

### Effect of time since stroke, gender, intracortical volume, age, and lesion volume

**Figure 3:**
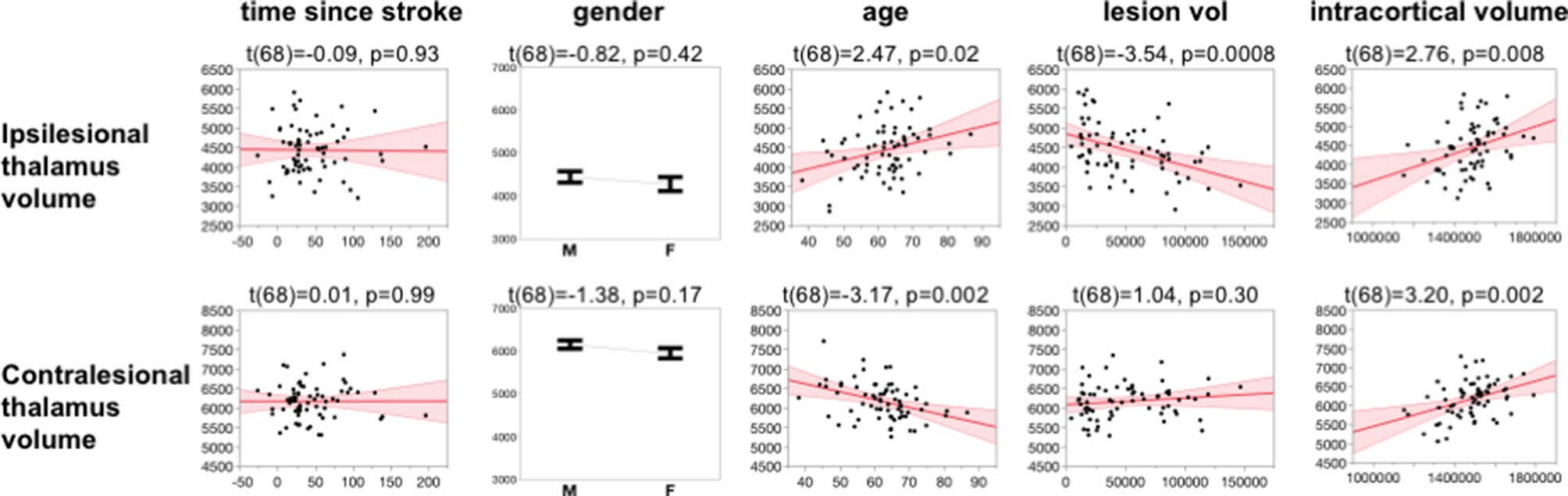
Modeling results of ipsilesional and contralesional thalamus volume. T-statistic and probability are shown above each plot.

The whole model for ipsilesional thalamus volume was significant (F(10,58) = 3.05, p = 0.004). Subsequent t-tests revealed a significant effect for intracortical volume (t(68) = 2.76, p = 0.008), age (t(68) = 2.47, p = 0.02), lesion volume (t(68) = − 3.54, p = 0.0008), and cross-term age*time since stroke (t(68) = 2.46, p = 0.02). Time since stroke (t(68) = − 0.09, p = 0.93) nor gender (t(68) = − 0.82, p = 0.42) were significantly related to in ipsilesional thalamus volume with this model. The whole model fit for contralesional thalamus volume was also significant (F(10,58) = 7.04, p < 0.0001). Subsequent t-tests revealed significant effect for intracortical volume (t(68) = 3.2, p = 0.002) and age (t = − 3.17, p = 0.002). Although adding both gender and intracranial volume may lead to multicollinearity in the model, the variance inflation factor for each variable was below 3 and therefore does not warrant corrective measures. A graphical summary of the effects can be seen in Fig. 3.

## Discussion

In this cross-sectional pilot study of chronic stroke participants, we tested five demographic factors that may have influence on ipsi-lesional and contra-lesional whole thalamus volume: (1) time since stroke, (2) gender, (3) intracortical volume, (4) age, and (5) lesion volume. We showed that thalamus volume in the chronic stages of stroke is related to intracortical volume, age, and lesion volume. These findings will require replication in the field.

We found that time since stroke does not describe thalamus volume in either ipsi- or contralesional hemispheres, which was unexpected as previous longitudinal studies have identified a significant negative longitudinal relationship in the acute and subacute stages^4,5^. Thus, hypothesis 1 was not confirmed. There could be two reasons for this negative finding in our study: (1) structural thalamus changes due to loss of input/output may be limited to the acute and subacute stages, or (2) the thalamus changes in the chronic stage related to time post onset are too subtle to study using current MRI techniques or in a cross-sectional manner. Additional study is warranted to validate these findings in a larger study cohort. Interestingly, this particular cohort showed that ipsilesional thalamus volume had a significant relationship with the age*time since stroke interaction term. This may be because older chronic stroke participants may have a longer time since stroke, while younger participants have a shorter time since stroke, and may be a product of the cross-sectional nature of this dataset.

There was no significant effect of gender in thalamus volume. Thus, Hypothesis 2 was not confirmed. Instead, the dominant and significant effect was with intracranial volume, which showed that a larger head size corresponds to a larger thalamus volume, regardless of ipsi- or contralateral placement. It is well accepted that males, on average, have larger head sizes than females, which is the case in our dataset as well. Although adding two correlated variables into the model to describe thalamus volume could lead to multicollinearity, no correction was warranted due to the small variance inflation factor of each variable. Thus, intracranial volume may be a more important factor to consider in modeling volumetrics than gender and may have statistical advantages in modeling and prediction due to its continuous nature.

As expected, the contra-lesional thalamus volume has a negative relationship with age. Such findings have previously been identified in healthy aging cohorts in both hemispheres^7,8^. Interestingly, the ipsi-lesional thalamus volume has a significantly positive relationship with age. Thus, Hypothesis 3 was only partially confirmed. It is unclear why the ipsi-lesional thalamus is larger in older chronic stroke participants compared to younger stroke participants. It may be that the longitudinal trajectory in thalamus decline differs in young and old, and that the cross-sectional nature of this dataset cannot adequately describe the effects of age-by-disease interaction in this manner. Importantly, the finding that the contralesional has a negative relationship with age and that the ipsilesional thalamus has a positive relationship with age requires replication and further scrutiny.

Finally, we showed that the ipsilesional thalamus volume has a significant negative relationship with lesion volume. This is likely due to loss of input or output into the thalamus of the lesioned hemisphere but remains to be tested as the quantification of structural or functional connections to the thalamus are beyond the scope of this work. In line with previous work, we found that the contralesional thalamus volume does not have a relationship with lesion volume. Thus, Hypothesis 4 was confirmed.

This pilot study had limitations and its findings should be replicated in a larger and preferably longitudinal cohort. First, although previous evidence points to a similarity in trajectories quantified from longitudinal and cross-sectional data in brain volume decline with healthy aging^16^, such similarities may not hold for stroke related changes. This may be the case in our lack of relationship between thalamus volume and time since stroke. Second, the retrospective nature of this study precluded acquisition of consistent demographic/functional descriptors such as NIH stroke scale and comorbidities across all participants and represents a major limitation. NIHSS assessments describe the stroke severity and are an important descriptor of stroke recovery and outcome. Further, Comorbidities such as diabetes, high blood pressure, history of smoking, heart disease, and other vascular risk factors stemming from lifestyle factors could cause small-vessel disease^17,18^ and reduce blood supply to the thalamus, in turn influencing the thalamus volume. The complex interplay of different comorbidities on stroke outcome and recovery is thought to influence the effect of neuroprotective drugs and their outcome in clinical trials^19^. Future studies identifying volumetric brain difference should also take these factors into account, including dietary and exercise habits, and may benefit from using large well-characterized datasets^20,21^. Finally, this study falls short of relating the thalamus volume to functional outcomes. That is likely the next important step to identify if the thalamus volume is relevant in predicting functional outcomes or rehabilitation potential after stroke.

In conclusion, we identified important demographic factors that influence the ipsi-lesional and contra-lesional thalamus volume, including intracortical volume, age, and lesion volume in a relatively small cross-sectional cohort of chronic stroke participants. The ipsi- and contra-lesional thalamus have unique relationships with age and requires further exploration.
